# Challenges and barriers to health care and overall health in older residents of Alaska: evidence from a national survey

**DOI:** 10.3402/ijch.v75.30348

**Published:** 2016-04-06

**Authors:** Julia D. Foutz, Steven A. Cohen, Sarah K. Cook

**Affiliations:** 1 Division of Epidemiology, Department of Family Medicine and Population Health, Virginia Commonwealth University, Richmond, VA, USA; 2 Center on Society and Health, Department of Family Medicine and Population Health, Virginia Commonwealth University, Richmond, VA, USA

**Keywords:** older adults, health care, Alaska

## Abstract

**Background:**

From 1970 to 2010, the Alaskan population increased from 302,583 to 698,473. During that time, the growth rate of Alaskan seniors (65+) was 4 times higher than their national counterparts. Ageing in Alaska requires confronting unique environmental, sociodemographic and infrastructural challenges, including an extreme climate, geographical isolation and less developed health care infrastructure compared to the continental US.

**Objective:**

The objective of this analysis is to compare the health needs of Alaskan seniors to those in the continental US.

**Design:**

We abstracted 315,161 records of individuals age 65+ from the 2013 and 2014 Behavioral Risk Factor Surveillance System, of which 1,852 were residents of Alaska. To compare residents of Alaska to residents of the 48 contiguous states we used generalized linear models which allowed us to adjust for demographic differences and survey weighting procedures. We examined 3 primary outcomes – general health status, health care coverage status and length of time since last routine check-up.

**Results:**

Alaskan seniors were 59% less likely to have had a routine check-up in the past year and 12% less likely to report excellent health status than comparable seniors in the contiguous US.

**Conclusions:**

Given the growth rate of Alaskan seniors and inherent health care challenges this vulnerable population faces, future research should examine the specific pathways through which these disparities occur and inform policies to ensure that all US seniors, regardless of geographical location, have access to high-quality health services.

Alaska has undergone rapid sociodemographic changes over the past 50 years, resulting in an increasingly diverse and urban population, as well as a growing number of adults over the age of 65 (1). The number of Americans aged 65 and older will increase roughly 38% in the next 10 years from approximately 48 million to nearly 66 million (2). In Alaska, this growth is projected to be even more drastic. The Alaska Department of Labor predicts that the number of Alaska residents aged 65 and older will more than double from 63,832 people in 2012 to 140,340 people in 2042, despite historically having a younger population than the rest of the United States (US) (3). An increase in the elderly population in Alaska means a dramatic increase in the number of people requiring medical care, long-term health services and other health-related services, such as informal caregiving and assistance with activities of daily living (4).

In addition to a growing elderly population, the rapid migration of younger family members from rural to urban communities are having direct impacts on the evolving notions of successful ageing for Alaska Native people (5). Such migration patterns are related to the increased educational, employment and housing opportunities that exist in urban settings (6). The departure of American Indians and Alaska Native people from reservations often results in a loss of access to health care, historically provided by the Indian Health Service (IHS) (7), due to lack of these services in urban areas outside of reservations. As this population moves to urban areas away from their home reservations, they cannot access IHS services, forcing them to rely on other sources of coverage or become uninsured (8). Alaska Native people make up about 17% of the Alaskan population (3). However, due to the passage of the Alaska Native Claims Settlement Act of 1971, the vast majority of Alaska Natives living in Alaska do not reside on reservations (9).

That said, the geography and extreme climate of Alaska also limit access to care. Alaska is the largest state in the United States, with 1,518,800 square kilometres (km) of territory, yet despite its size is the 4th least-populous state with a low population density of 1.1 persons per square mile. In 2000, Alaskan's population was roughly 66% urban with over half of the state's population concentrated in Anchorage (10). However, approximately 25% of all Alaskans and 46% of Alaska Natives live in communities of less than 1,000 people. Nearly one quarter of the state's population lives in towns and villages that are reachable only by boat or aircraft. Approximately 75% of Alaskan communities are not connected by road to another community with a hospital. Air travel within Alaska is expensive, and severe weather often limits air travel, causing delays in obtaining care (11).

High rates of poverty, increasing effects of climate change, geographical isolation and limited access to health care pose additional public health challenges to the health of older adults living in Alaska (12). Although these problems affect the entire population, older adults in Alaska tend to be disproportionately impacted due to pre-existing vulnerabilities (13). Health disparities are also apparent among the Alaska Native population and rural residents of the state, with older members of these populations impacted the most (10). With the number of older adults living in Alaska expected to increase rapidly in the next 2 decades, developing a better understanding of health care access and preventative health behaviours of older adults will be an important step towards meeting the needs of this growing population.

Although many of these issues – poverty, climate change, geographical isolation and health disparities in older adults – are well documented, few studies (14,15) have assessed how these issues impact the health of older adults in Alaska, and none have directly compared these issues in Alaska to the contiguous US. Furthermore, few studies to date have assessed the specific health factors and health behaviours that may be exacerbated in Alaskan older adults compared to older adults living in the contiguous US. Therefore, this study seeks to examine health care access and preventative health behaviours of older adults in Alaska compared to older adults in the rest of the contiguous US.

## Methods

### Data

Data were obtained from the 2013 and 2014 Behavioral Risk Factor Surveillance System (BRFSS), a national cross-sectional telephone-based survey of adults aged 18 or older (16). BRFSS is conducted annually and administered by state health departments with oversight from the Centers for Disease Control and Prevention, reaching over 400,000 respondents across the United States and its territories. This analysis is limited to respondents aged 65 and older and includes respondents living in the 48 contiguous US states and the District of Columbia (N=315,161).

### Outcome

To compare health care–seeking behaviours and health status between residents of Alaska and residents of the contiguous 48 states, 3 outcome measures were used in this study: (a) health insurance status, (b) length of time since last routine check-up and (c) self-reported overall health. To determine health insurance status, respondents were asked if they had any health care coverage, including health insurance, prepaid plans such as HMOs or government plans such as Medicare or IHS. This was then combined into a dichotomous (yes/no) variable for analysis. To determine length of time since last routine check-up, respondents reported how long it had been since they last visited a doctor, which was then combined into a dichotomous (<12 months/≥12 months) variable for analysis. Self-reported overall health was based on responses to the question, “Would you say your health in general is excellent, very good, good, fair, or poor?” which was assessed as a 5-level categorical variable.

### Exposure

The primary exposure measure was each respondent's state of residence, dichotomized as Alaskan versus non-Alaskan (the rest of the United States). The non-Alaskan group was used as the reference group for analysis.

### Covariates

Categorical covariates used in this analysis include age, sex, race/ethnicity, annual household income and highest education attained. Body mass index (BMI) calculated from self-reported height and weight was used as a continuous covariate in the statistical analysis.

### Analysis

We examined differences in demographic characteristics for respondents living in Alaska compared to respondents living in the rest of the United States using chi-square tests of independence for categorical variables and *t* tests for the continuous BMI variable.

Logistic regression was used to assess the relationship between Alaskan and non-Alaskan residence and health care–seeking behaviours and population health status. Separate logistic regression models were fit for health insurance status and length of time since last routine check-up. An ordinal logistic regression model was fit for self-reported overall health to allow the use of the variable in its original, 5-level ordinal form. Crude and adjusted models were examined for all 3 outcomes. In addition to the primary outcome variable, fully adjusted models included age, sex, race/ethnicity, income, education and BMI as covariates. Point estimates and standard errors were analysed using survey procedures in SAS (v9.4) to account for the complex sampling design.

## Results

Less than 1% (N=1,852) of respondents resided in Alaska at the time of the survey (Table I). Respondents living in Alaska were more likely to belong to younger age groups than respondents living in the rest of the US (X^2^=59.42, p<0.001). Respondents living in Alaska were more likely to be female (X^2^=11.12, p≤0.001) and more likely to identify as American Indian or Alaska Native race/ethnicity (X^2^=809.75, p<0.001) relative to non-Alaskans. Respondents living in Alaska had higher incomes (X^2^=102.61, p<0.001) and were more likely to have some college or trade school (X^2^=37.50, p<0.001) than respondents living in the rest of the United States. Residents of Alaska were less likely to have Medicare (X^2^=4.39, p=0.036) or to have had a check-up in the past year (X^2^=153.55, p<0.001), but were more likely to self-report better overall health (X^2^=13.27, p=0.010) than non-residents of Alaska. Overall, there were no significant differences in health insurance status and BMI between Alaska and non-Alaska residents.

**Table I:** Alaska compared to the rest of the United States: Demographics of adults 60 and older

	Alaska	United States	
		
N=315,161	1,852	313,309	X^2^ or T, p value
Age			59.42, <0.001
65–69	41.04	32.49	
70–74	26.47	24.62	
75–79	18.60	19.78	
≥80	13.89	23.10	
Sex			11.12, <0.001
Male	49.16	43.81	
Female	50.84	56.19	
Race/ethnicity			809.75, <0.001
White	76.13	79.37	
Black	2.28	9.11	
Asian/NHOPI	3.19	2.54	
Hispanic	3.08	6.89	
Am Ind/AK Native	11.52	0.79	
Other/2+ races	3.80	1.30	
Income			102.61, <0.001
<$15,000	10.17	12.20	
$15,000 to <$25,000	14.12	22.50	
$25,000 to <$35,000	9.52	15.07	
$35,000 to <$50,000	17.92	16.85	
≥$50,000	48.26	33.39	
Education			51.67, <0.001
<High school	12.24	17.01	
High school graduate	25.14	31.74	
Some college/trade	37.50	28.20	
College/trade graduate	25.12	23.05	
BMI (mean)	28.09	27.58	−3.70, <0.001
Health insurance			<0.001, 0.9762
Yes	98.37	98.38	
No	1.63	1.62	
Check-up in past year			153.55, <0.001
Yes	73.68	87.78	
No	26.32	12.22	
Self-reported health			13.27, 0.010
Excellent	13.20	12.42	
Very good	32.97	28.37	
Good	31.24	33.49	
Fair	15.14	18.11	
Poor	7.45	7.62	
Medicare			4.39, 0.036
Yes	93.09	94.53	
No	6.911	5.47	

Am Ind=American Indians; AK Native=Alaska Native.

Values are weighted percent unless otherwise noted.

Table II shows the results of 3 sets of logistic regression models, unadjusted and adjusted. Models 1 and 2 predict having health insurance coverage by US state residence. In both the unadjusted and adjusted models, Alaskan residence was not associated with the decreased likelihood of having health insurance coverage (OR (odds ratio): 0.99, 95% CI (confidence interval): 0.59–1.67; OR: 0.86, 95% CI: 0.48–1.54, respectively). Overall, respondents most likely to have health insurance coverage included older age groups, women, non-Hispanic Whites, annual income greater than $15,000 and attainment of high school education of higher.

**Table II:** Logistic regression of preventative health behaviours in Alaskans vs. non-Alaskans aged 65 and older

	Healthcare coverage		Check-up within the past year		Better self-reported overall health	
			
	Odds ratio (95% CI)	p value	Odds ratio (95% CI)	p value	Odds ratio (95% CI)	p value
	
	Model 1: Unadjusted		Model 3: Unadjusted		Model 5: Unadjusted	
State		0.976		<0.001		0.019
Non-Alaskans	1.0 (ref)		1.0 (ref)		1.0 (ref)	
Alaskans	0.99 (0.59–1.67)		0.39 (0.33–0.46)		0.87 (0.77–0.98)	

	Model 2: Adjusted		Model 4: Adjusted		Model 6: Adjusted	

State		0.610		<0.001		0.056
Non-Alaskans	1.0 (ref)		1.0 (ref)		1.0 (ref)	
Alaskans	0.86 (0.48–1.54)		0.41 (0.34–0.48)		0.88 (0.78–1.00)	
Age		<0.001		<0.001		0.717
65–69	1.0 (ref)		1.0 (ref)		1.0 (ref)	
70–74	1.26 (1.02–1.57)		1.26 (1.18–1.34)		1.00 (0.96–1.04)	
75–79	1.66 (1.29–2.12)		1.52 (1.41–1.64)		0.98 (0.94–1.03)	
≥80	1.33 (1.07–1.65)		1.83 (1.70–1.97)		1.01 (0.97–1.05)	
Sex		<0.001		<0.001		<0.001
Male	1.0 (ref)		1.0 (ref)		1.0 (ref)	
Female	1.66 (1.41–1.96)		1.16 (1.10–1.22)		0.93 (0.90–0.96)	
Race/ethnicity		<0.001		<0.001		<0.001
White	1.0 (ref)		1.0 (ref)		1.0 (ref)	
Black	0.59 (0.47–0.74)		1.80 (1.59–2.04)		1.20 (1.13–1.27)	
Asian/NHOPI	0.31 (0.17–0.55)		1.02 (0.76–1.35)		1.12 (0.95–1.32)	
Hispanic	0.39 (0.30–0.51)		1.05 (0.91–1.22)		1.35 (1.25–1.47)	
Am Ind/AK Native	0.70 (0.44–1.11)		0.86 (0.68–1.09)		1.10 (0.94–1.28)	
Other/2+ Races	0.74 (0.49–1.12)		0.80 (0.65–0.99)		1.24 (1.10–1.39)	
Income		<0.001		<0.001		<0.001
<$15,000	1.0 (ref)		1.0 (ref)		1.0 (ref)	
$15,000 to <$25,000	1.22 (0.98–1.53)		1.05 (0.96–1.16)		0.93 (0.89–0.98)	
$25,000 to <$35,000	1.37 (1.02–1.84)		1.13 (1.01–1.25)		0.88 (0.84–0.93)	
$35,000 to <$50,000	2.08 (1.54–2.81)		1.20 (1.09–1.33)		0.79 (0.74–0.84)	
≥$50,000	4.39 (3.25–5.92)		1.29 (1.17–1.43)		0.82 (0.77–0.87)	
Education		<0.001		0.014		<0.001
<High school	1.0 (ref)		1.0 (ref)		1.0 (ref)	
High school grad	1.54 (1.24–1.90)		1.14 (1.04–1.25)		0.89 (0.84–0.93)	
Some college/TS	1.80 (1.40–2.32)		1.06 (0.96–1.17)		0.86 (0.82–0.91)	
College/TS grad	2.42 (1.81–3.24)		1.06 (0.96–1.17)		0.92 (0.87–0.97)	
BMI	1.01 (0.99–1.03)	0.257	1.03 (1.03–1.04)	<0.001	1.00 (1.00–1.00)	0.245

Am Ind=American Indians; AK Native=Alaska Native.

Models 3 and 4 predict having been to a routine check-up with a doctor within the past year by US state residence. Alaska residents were 59% less likely to have had a check-up within the past year, after controlling for other factors (OR: 0.41, 95% CI: 0.34–0.48). Older age groups, women, respondents who identify as non-Hispanic Black, respondents who report an income level ≥$35,000, and respondents with higher BMIs were also significantly more likely to have been to a doctor within the past year.

Models 5 and 6 predict higher overall self-reported health by US state residence. In the unadjusted model, Alaska residents were significantly less likely to report better health than non-Alaska residents (OR: 0.87, 95% CI: 0.77–0.98). Although not statistically significant, residents of Alaska also reported poorer overall health than non-Alaskan residents in the adjusted model (OR: 0.88, 95% CI: 0.78–1.00). Women, respondents who identify as non-Hispanic White respondents who report an income ≥$15,000 and respondents with a high school education or higher were more likely to report poorer overall health. As an ad hoc analysis, we also added an interaction term of American Indians/Alaska natives (AI/AN) and Alaska status to determine if Natives in Alaska were more or less likely to have insurance, see a physician, or be in better health, but the term was not statistically significant for any of the 3 models.

## Discussion

The findings of this study suggest that, even after accounting for potential sociodemographic confounders, older adults living in Alaska are less than half as likely as older adults from the contiguous United States to have had a routine medical check-up within the past year, yet are less likely to report better overall health, although that association became less significant after controlling for confounders. These findings suggest that, despite universal health insurance coverage eligibility for older adults aged 65+ through Medicare, many older Alaskans do not get regular medical check-ups. One key challenge that needs further exploration is how the health care community can better connect this vulnerable population to better health care access.

The potential explanations for the discrepancies in receiving a check-up in the past year comparing Alaskan to non-Alaskan older adults are numerous. Alaskan older adults may not seek health care due to distance to provider or other factors (16). Prior studies have suggested that health care utilization is adversely affected by long travel times, particularly if the distance between one's residence and provider is greater than 32 km (20 miles) away (17,18). Some state health departments have implemented a standard in which rural residents would not need to travel more than 30 min to see a primary care physician (19). The 30-min standard may not be feasible in Alaska due to the geographical layout of villages and facilities across the state, given the extreme remoteness and rurality of much of the state. Older adults living in those most remote areas may not have adequate access to care as a result.

Compounding the disparities observed in Alaskan older adults are possible racial and ethnic disparities between AI/AN and other racial and ethnic groups (20). A key finding of our study is that regardless of geographical location, AI/AN adults were less likely than non-Hispanic Whites to have had a check-up in the last year, and less likely to have better self-reported health, even after controlling for place of residence (Alaska vs. contiguous US) and all other sociodemographic factors. Although research on such racial disparities is somewhat limited, one study comparing AI/AN adults living in Alaska to AI/AN adults living in the contiguous US suggested that AI/AN adults living in Alaska actually had higher rates of some preventive health behaviours and screening procedures, including Pap smear testing, mammography and faecal occult blood testing for colorectal cancer (21). However, that study focused primarily on comparing AI/AN adults living in Alaska to non-Hispanic Whites living in the contiguous US and did not focus exclusively on older adult health. Other studies have suggested notable disparities in health services access, insurance coverage and health-seeking behaviours between AI/AN populations and Whites (5,6). However, much of this research focuses on examining AI/AN disparities across the United States. The relationships between health behaviours and race and ethnicity may vary by place of residence (Alaska vs. non-Alaska) (21). Therefore, quantifying and addressing AI/AN health disparities, particularly within Alaska, is an important issue that should be addressed further in future studies (4).

The findings of this study are subject to several important limitations and caveats. First, this study uses data from a large, national, telephone-based survey of the US population. As such, the coverage of the survey itself may be biased by rurality inherent to Alaska. Alaska residents living in highly remote areas may not have access to landline or cellular telephones and would not be eligible to be included in this survey. Second, data quality may be somewhat limited by the self-reported nature of the survey data. Third, as the data are cross-sectional, causality cannot be ascertained. Next, this study compared residents of Alaskan non-Alaskans residents of other states and did not examine potential key geographical and sociodemographic factors within Alaska itself and their potential impacts on health. The sociodemographic composition of the Alaska population is diverse. Furthermore, there may be additional disparities in health and potential drivers of those health disparities (e.g. cost, supply of health care workers and insurance reimbursements) that may be masked when comparing Alaska as a whole to the rest of the United States. Future research should examine these factors within the state that may influence these and other health-related conditions. Lastly, this study examined only 3 outcomes: health care coverage, check-ups in the last year and self-reported health. Health and health care–seeking behaviours are far more complex than these 3 outcomes alone. Further research should explore any of the numerous other aspects of health and health care–seeking behaviours that contribute more fully to population health.

Despite these limitations, this is among the first such studies to highlight several health care disparities between older adults living in Alaska and those living in the contiguous US using a large, nationally representative sample of residents, controlling for key socioeconomic and demographic characteristics. These preliminary findings suggest that, even after controlling for those socioeconomic and demographic characteristics that often drive health disparities, there remained a significant difference in preventive health behaviours between older adults living in Alaska to those living outside Alaska and borderline significant (p=0.056) difference in overall health. Such results have potentially important implications to initiate deeper research into why these disparities occur and what can be done to ameliorate them. As the rapid growth of the older population in Alaska outpaces the growth of the older population across the rest of the United States, the need to quantify and address these disparities is increasing. Additional research is needed to understand the underlying mechanisms for these disparities to ensure equitable health care access for all older adults across all 50 states.
